# Edaravone efficacy in amyotrophic lateral sclerosis with reduced forced vital capacity: Post-hoc analysis of Study 19 (MCI186-19) [clinical trial NCT01492686]

**DOI:** 10.1371/journal.pone.0258614

**Published:** 2022-06-14

**Authors:** Benjamin Rix Brooks, Terry Heiman-Patterson, Martina Wiedau-Pazos, Shawn Liu, Jeffrey Zhang, Stephen Apple

**Affiliations:** 1 Atrium Health Neurosciences Institute, Carolinas Medical Center, University of North Carolina School of Medicine–Charlotte Campus, Charlotte, North Carolina, United States of America; 2 Lewis Katz School of Medicine, Temple University, Philadelphia, Pennsylvania, United States of America; 3 David Geffen School of Medicine, University of California, Los Angeles, Los Angeles, California, United States of America; 4 Mitsubishi Tanabe Pharma Development America, Jersey City, New Jersey, United States of America; 5 Princeton Pharmatech, West Windsor, New Jersey, United States of America; 6 Mitsubishi Tanabe Pharma America, Inc., Jersey City, New Jersey, United States of America; First Hospital of Jilin University, CHINA

## Abstract

**Background:**

Edaravone slowed the rate of functional decline in subjects with amyotrophic lateral sclerosis (ALS) in phase 3 study MCI186-19 (Study 19). One of the Study 19 inclusion criteria was forced vital capacity (FVC) ≥80% of predicted (≥80%p). Therefore, the study provided no information on edaravone efficacy in subjects with FVC <80%p. In Study 19, 24-week, double-blind treatment was followed by open-label treatment where all subjects received edaravone. At 24 weeks, some subjects had FVC <80%p (FVC_24_ <80%p). This allowed for post-hoc assessment of the effects of edaravone in subgroups of subjects with FVC_24_ ≥80%p vs <80%p.

**Objective:**

To address the question of the efficacy of edaravone in ALS patients with FVC <80%p.

**Methods:**

Post-hoc analysis of Study 19 comparing edaravone efficacy at week 48 in subjects with FVC_24_ ≥80%p vs <80%p.

**Results:**

With edaravone treatment, subjects in both the FVC_24_ ≥80%p and the FVC_24_ <80%p subgroups experienced a reduction in ALS Functional Rating Scale-Revised (ALSFRS-R) score loss vs placebo subjects through week 48. For the FVC_24_ ≥80%p subgroup, the changes in ALSFRS-R scores from baseline to week 48 were −7.63 for edaravone-edaravone vs −9.69 for placebo-edaravone, a difference of 2.05 (*P* = .034; 95% CI: 0.16, 3.94). For the FVC_24_ <80%p subgroup, the changes in ALSFRS-R scores from baseline to week 48 were −10.26 for edaravone-edaravone vs −15.20 for placebo-edaravone, a difference of 4.94 (*P* = .0038; 95% CI: 1.64, 8.25). Linear regression analysis indicated that, in the FVC_24_ <80%p subgroup, there was a notable change in the slope of the ALSFRS-R score-vs-time graph after the start of edaravone treatment.

**Conclusion:**

ALS subjects in the Study 19 placebo arm had a slowing in disease progression, even when edaravone was added with an FVC of <80%p prior to starting edaravone. A randomized, placebo-controlled study is needed to validate these post-hoc findings.

## Introduction

Amyotrophic lateral sclerosis (ALS) is a progressive and fatal neuromuscular disease, characterized by the degeneration of nerve cells of the brain and spinal cord, predominantly upper and lower motor neurons [1]. The lifespan for patients with ALS is typically 3 to 5 years from the time of disease onset, and the mortality rate is 50% within 30 months of symptom onset [2]. The majority of patients succumb to respiratory failure [1, 3, 4]. Therefore, monitoring for respiratory function is critical to both track disease progression and to inform decision-making on when to initiate appropriate respiratory support [3–6].

One of the main surrogate measures for respiratory function in patients with ALS is forced vital capacity (FVC) [3–5]. There is a linear correlation between FVC and disease progression and survival [7, 8]. In the assessment of patients with ALS, those with an FVC at or above 80% of predicted (80%p) are considered to have normal respiratory function; FVC <50%p usually indicates the need for respiratory support [5, 9].

Radicava^®^ (edaravone) is approved by the United States Food and Drug Administration (FDA) for the treatment of ALS and has been shown to slow the rate of functional decline [10]. The FDA approval was based on the outcomes from edaravone Study 19 (MCI186-19; clinicaltrials.org, NCT01492686), which was a 24-week, randomized, double-blind, placebo-controlled study conducted in Japan [11]. An earlier phase 3 study of edaravone (study MCI186-16) suggested a benefit with edaravone treatment, although the difference between study arms was not significant; it was believed to be due to heterogeneity in the study population and a notable proportion of subjects with slow disease progression [12, 13]. Study 19 employed a strategic, enrichment study design in order to measure a treatment effect in a 6-month time frame, as indicated by the score on the ALS Functional Rating Scale-Revised (ALSFRS-R) [2, 14–16]. This enrichment strategy enrolled subjects with relatively high functionality at baseline [2, 16]. With regard to respiratory function, all subjects were required to have a score of 4 on the respiratory items of the ALSFRS-R (item numbers 10–12: dyspnea, orthopnea, and respiratory insufficiency), and an FVC ≥80%p, thereby helping to select for subjects with good respiratory function at the start of the trial [11]. The enrichment strategy also ensured a subject population that would undergo adequate progression, which is needed in order to evaluate a treatment effect [2, 16].

In Study 19, edaravone treatment was associated with a significant reduction in the rate of decline in ALSFRS-R score [11]. During the 12-week double-blind treatment period, the least squares (LS) mean (± standard error) change in ALSFRS-R score was −5.01 ± 0.64 in the edaravone group vs −7.50 ± 0.66 in the placebo group (LS mean difference between groups of 2.49 ± 0.76; *P* = .0013) [11]. In addition, during the double-blind period, there was less of a decline in FVC with edaravone than with placebo, although the difference was not statistically significant; the LS mean change from baseline in FVC was −15.61 ± 2.41%p in the edaravone group vs −20.40 ± 2.48%p in the placebo group (LS mean difference between groups of 4.78 ± 2.84; *P* = .0942) [11]. In Study 19, the 24-week, double-blind treatment period was followed by a 24-week, open-label, active treatment period in which all subjects received edaravone treatment. An analysis of FVC during the entire 48-week time frame of the study revealed that there was a significantly lower reduction in FVC in subjects originally enrolled in the edaravone treatment arm vs those in the placebo arm; the LS mean change from baseline FVC was −28.24 ± 3.52 in the edaravone-edaravone group vs −40.12 ± 3.72 in the placebo-edaravone group (LS mean difference between groups, 11.88 ± 5.05; *P* = .0207) [17]. In addition, edaravone was associated with a lower rate of discontinuations during the study (8 in the placebo group vs 2 in the edaravone group), most of which were related to worsening disease, including respiratory decline [11, 17].

Whether the results of Study 19 are generalizable to real-world clinical practice has been questioned by both clinicians and payors [2] and might be informed by post-hoc analysis of Study 19. Moreover, as one of the Study 19 inclusion criteria was an FVC ≥80%p, questions have arisen regarding the efficacy of edaravone in subjects with FVC <80%p. To address this issue, a post-hoc analysis was conducted to evaluate the effect of edaravone in subgroups of subjects from Study 19 at 48 weeks, differentiated by their FVC values at the end of the 24-week double-blind treatment period (FVC_24_ ≥80%p vs FVC_24_ <80%p).

## Methods

### Study 19 (MCI186-19) study design

**Fig 1:**
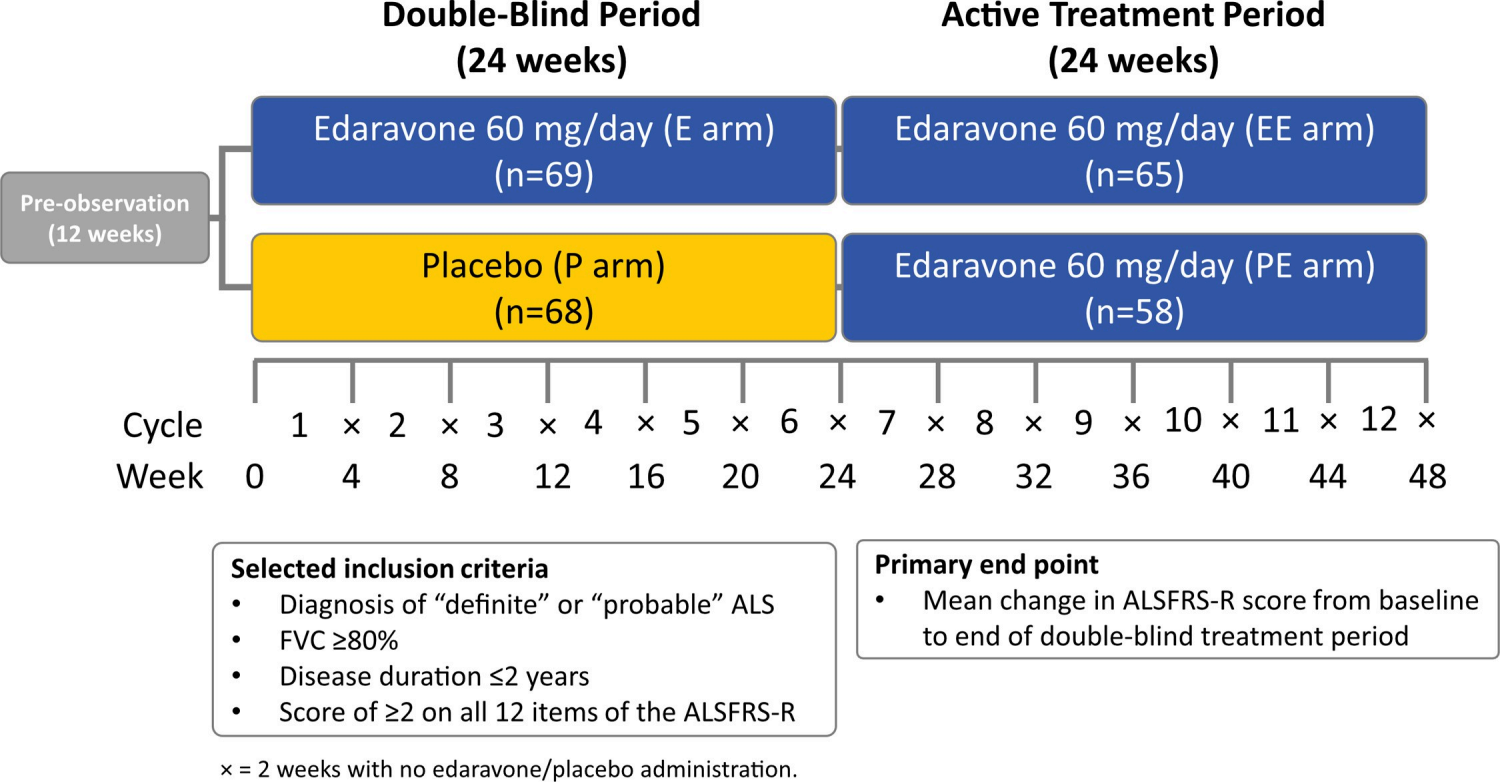
Study design for edaravone Study 19. After a 12-week pre-observation period, eligible subjects were randomized to receive either edaravone or placebo during a 24-week, double-blind period. After the double-blind period, subjects received open-label edaravone for 24 weeks. The diagram shows the treatment cycle and corresponding weeks, along with selected inclusion criteria and the primary end point. ALS, amyotrophic lateral sclerosis; ALSFRS-R, Amyotrophic Lateral Sclerosis Functional Rating Scale-Revised; E, edaravone; FVC, forced vital capacity; P, placebo.

Study 19 was a randomized, double-blind, parallel-group, placebo-controlled study (**Fig 1**) [11]. The details of study methodology, subject selection (described below, under ***Participants***), ethical study conduct, end points, and prospective statistical analyses for the 24-week double-blind period and 24-week open-label active treatment period have been previously described in detail (clinicaltrials.org: NCT01492686) [11, 18].

Subjects eligible to enter the 24-week, double-blind period were identified after a 12-week observation period. Those with a decrease of 1 to 4 points in the ALSFRS-R score during observation were deemed eligible and were randomly assigned 1:1 to edaravone or placebo for 24 weeks (6 cycles). A 24-week, open-label, active treatment period began at the end of cycle 6, at which time edaravone was administered to all subjects who rolled over from the double-blind phase, for an additional 24 weeks (6 cycles up to cycle 12). Edaravone was administered in a 60-mg dose via intravenous infusion over 60 minutes. Infusions were administered once per day for 14 days for the first treatment cycle and for 10 days of the 14-day treatment period for all subsequent cycles. Each treatment cycle was followed by a 14-day drug-free period. The primary efficacy end point of Study 19 was the change in ALSFRS-R score from baseline to the end of week 24. Secondary end points included the change in FVC, scores on the Modified Norris Scale (limb, bulbar, and total) and the ALS Assessment Questionnaire, the Japan ALS severity classification, and grip and pinch strength.

### Participants

Between November 28, 2011, and September 3, 2014, 213 subjects were screened, 137 of whom completed the observation period and were randomly assigned to receive edaravone (n = 69) or placebo (n = 68). Subjects who were eligible for Study 19 fulfilled the following criteria: (a) 20 to 75 years of age, (b) ALS of grade 1 or 2 in the Japan ALS Severity Classification, (c) scores of at least 2 points on all 12 items of ALSFRS-R, (d) FVC ≥80%p, (e) definite or probable ALS according to the El Escorial and revised Airlie House criteria, and (f) duration of disease from the first symptom (any ALS symptom) of 2 years or less [11]. Subjects were excluded before randomization if they had a score of 3 or less on ALSFRS-R items for dyspnea, orthopnea, or respiratory insufficiency; a history of spinal surgery after onset of ALS; or creatinine clearance of 50 mL/min or less. Initiation of riluzole after the start of the observation period was prohibited; however, subjects who had already been given riluzole could continue the medication, provided that the regimen remained unchanged. Study discontinuation occurred at subject request or if a subject was ineligible for the study, experienced an adverse event, required tracheotomy, required all-day respiratory support, or had worsening of ALS.

### Post-hoc assessment

For the current study, a post-hoc analysis was conducted to examine the changes in ALSFRS-R scores from baseline to week 24, week 24 to week 48, and baseline to week 48 among subjects in 2 subgroups based on their FVC values at week 24, FVC_24_ ≥80%p and FVC_24_ <80%p. Analyses were performed on data from observed cases at week 24 and week 48.

### Post-hoc analysis statistics

The post-hoc analysis population included 88 subjects who had a final data collection at the end of cycle 12 (51 subjects from the edaravone group and 37 subjects from the placebo group). Five subjects (2 from the edaravone group and 3 from the placebo group) had a final data collection at the end of cycle 11 (thus qualifying for inclusion as having completed the open-label treatment period) but did not have data for the end of cycle 12 and were therefore not included in the subsequent post-hoc analyses described in this article. For baseline demographic data, *P* values were calculated by using Student’s t-test for continuous variables and chi-square test for categorical variables. A mixed-effects model for repeated measures (MMRM) analysis, with an unstructured covariance matrix, was conducted on observed cases to determine the differences between the subgroup treatment arms in the change in ALSFRS-R score from baseline to week 24, from week 24 to week 48, and from baseline to week 48. Multiple linear regression analyses were performed with observed data to estimate the slopes of the scores for the treatment arms for the edaravone, placebo, edaravone-edaravone, and placebo-edaravone subjects in each subgroup. In addition, a linear mixed-effect model with fixed effect of linear slope of time, treatment, baseline value and interaction of time and treatment, and random effect of intercept was used to assess the differences in linear slopes. Data reflect LS mean differences in observed values between treatment arms and LS mean changes from baseline within each treatment arm. All analyses were conducted with SAS version 9.4.

## Results

### Subject disposition and baseline characteristics

**Fig 2:**
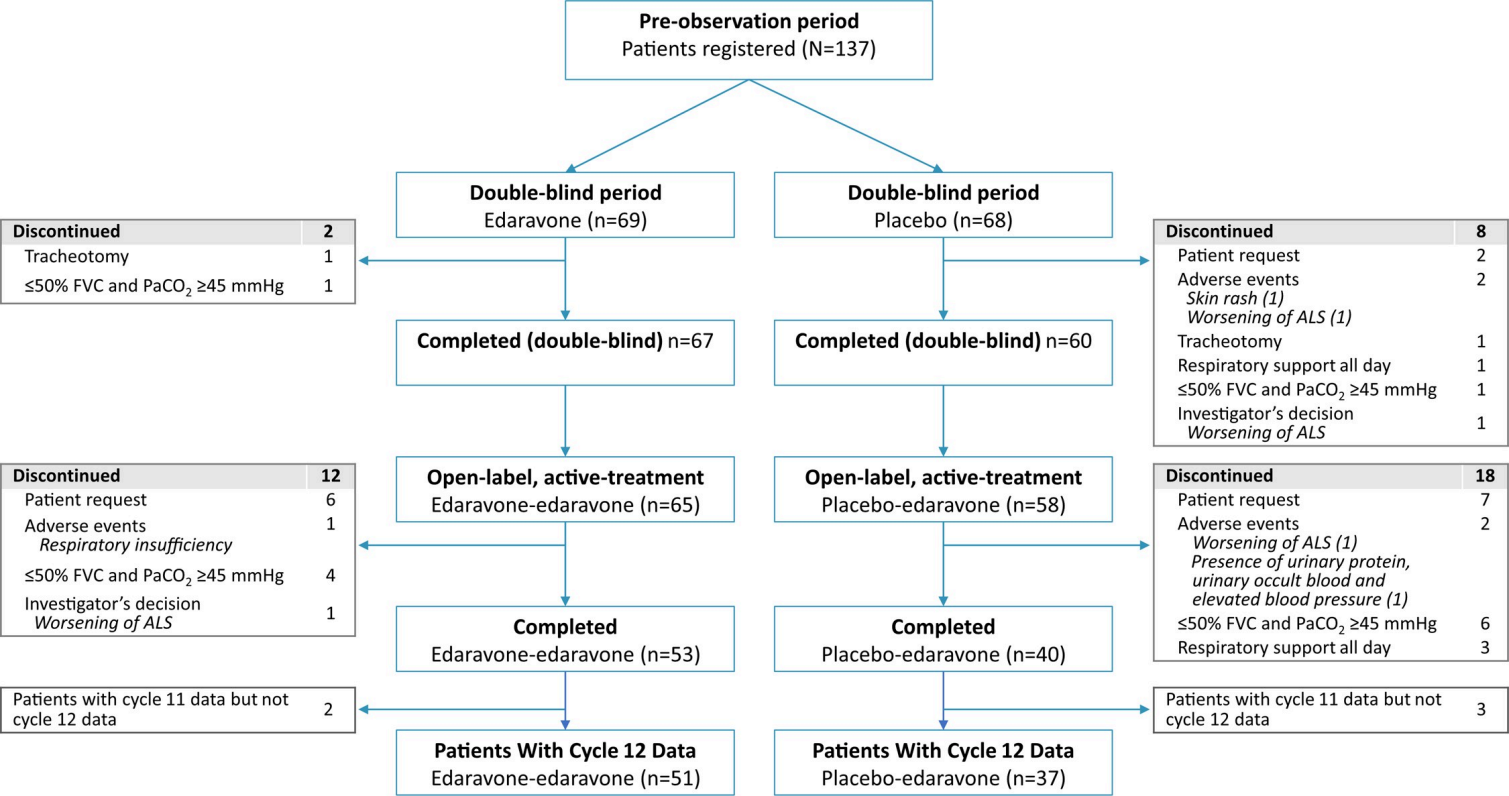
Subject disposition. The diagram shows subject disposition for the various stages of Study 19 and reasons for discontinuation. Note that for the overall study, subjects who returned for follow-up through cycle 11 were considered to have “completed” the study; however, a total of 5 subjects did not have data available for cycle 12 (2 subjects in the edaravone-edaravone group and 3 in the placebo-edaravone group, as indicated in the figure) and those subjects were excluded from the statistical analyses presented in this study. Adapted with permission [19]. ALS, amyotrophic lateral sclerosis; FVC, forced vital capacity (% of predicted); PaCO_2_, partial pressure of carbon dioxide in arterial blood.

A total of 137 subjects were initially randomized to receive either edaravone (n = 69) or placebo (n = 68) in the double-blind phase; 127 completed the double-blind period. During the double-blind treatment period, 2 edaravone and 8 placebo subjects discontinued treatment, with most of these subjects discontinuing due to disease progression (**Fig 2**). Of the 123 subjects continuing into the active-treatment period, 65 subjects were from the edaravone group (edaravone-edaravone) and 58 subjects were from the placebo group (placebo-edaravone). From these groups, 53 and 40 subjects completed the 24-week open-label treatment period, respectively. At the end of the open-label extension, there was a significant difference in the rate of discontinuation between groups: 23% discontinuation for subjects initially assigned to edaravone, contrasted with 41% for subjects initially assigned to placebo (*P* = .024).

Overall, at the start of the study, the demographics and baseline characteristics of subjects were well balanced between treatment groups, with numerical but nonsignificant differences for male sex and ALS severity (**Table 1**).

**Table 1:** Baseline demographics and clinical characteristics in Study 19 [11].

	Edaravone (n = 69)	Placebo (n = 68)	*P* value^a^
**Sex, n (%)**			0.5363
Male	38 (55)	41 (60)	
Female	31 (45)	27 (40)	
**Mean age (SD), y**	60.5 (10)	60.1 (10)	0.8111
**Mean duration of disease (SD), y**	1.13 (0.5)	1.06 (0.5)	0.8111
**Initial symptom, n (%)**			0.7129
Bulbar symptom	16 (23)	14 (21)	
Limb symptom	53 (77)	54 (79)	
**ALS diagnostic criteria, n (%)** ^**b**^			0.9169
Definite	28 (41)	27 (40)	
Probable	41 (59)	41 (60)	
**ALS severity, n (%)** ^**c**^			0.2748
Grade 1	22 (32)	16 (24)	
Grade 2	47 (68)	52 (76)	
**Mean ALSFRS-R score (SD)**			
Before observation period	43.6 (2.2)	43.5 (2.2)	0.8331
Baseline (end of 12 weeks observation)	41.9 (2.4)	41.8 (2.2)	0.8225
**Concomitant riluzole, n (%)**	63 (91)	62 (91)	0.9789

^a^Comparison between treatment groups.

^b^According to revised El Escorial criteria.

^c^According to Japan ALS severity classification (grade 1–5, with grade 5 being most severe).

ALS, amyotrophic lateral sclerosis; ALSFRS-R, Amyotrophic Lateral Sclerosis Functional Rating Scale-Revised; SD, standard deviation.

Reprinted from Lancet Neurol, 16, Writing Group; Edaravone (MCI-186) ALS 19 Study Group, Safety and efficacy of edaravone in well defined patients with amyotrophic lateral sclerosis: a randomised, double-blind, placebo-controlled trial., 505–12, 2017, with permission from Elsevier.

### Post-hoc analysis, baseline characteristics

For the post-hoc analysis, each treatment group was divided into 2 subgroups based on FVC_24_ (end of cycle 6) (). These 2 subgroups had comparable age, duration of disease, ALS diagnostic criteria, and concomitant use of riluzole (**Table 2**). However, the FVC_24_ <80% group had a higher proportion of females, a higher proportion of bulbar-onset subjects, a higher proportion of subjects with ALS severity grade 2, and a lower baseline ALSFRS-R score, suggesting that the subjects whose FVC fell to <80% by week 24 may have had more severe ALS at baseline than those who maintained FVC ≥80% through week 24 (**Table 2**). Within each subgroup, the treatment arms (ie, edaravone vs placebo) were well balanced for each baseline characteristic.

**Table 2:** Baseline demographics and clinical characteristics in FVC_24_ subgroups.

	FVC_24_ <80%p				FVC_24_ ≥80%p				*P* value^a^
	Edaravone (n = 28)	Placebo (n = 28)	FVC_24_ <80%p Total	*P* value^b^	Edaravone (n = 40)	Placebo (n = 33)	FVC24 ≥80%p Total	*P* value^b^	
			(n = 56)				(n = 73)		
**Sex, n (%)**				0.5920				0.6707	0.0182
Male	12 (43)	14 (50)	26 (46)		26 (65)	23 (70)	49 (67)		
Female	16 (57)	14 (50)	30 (54)		14 (35)	10 (30)	24 (33)		
**Mean age (SD), y**	61.3	60.5	60.9	0.7523	59.8	59.2	59.5	0.8100	0.4381
	(10.7)	(7.5)	(9)		(9.9)	(11.4)	(11)		
**Mean duration of disease (SD), y**	1.12	1.05	1.09	0.5568	1.14	1.10	1.12	0.7470	0.6529
	(0.5)	(0.4)	(0.4)		(0.5)	(0.5)	(0.5)		
**Initial symptom, n (%)**				0.4076				0.8085	<0.001
Bulbar symptom	12 (43)	9 (32)	21 (38)		3 (8)	2 (6)	5 (7)		
Limb symptom	16 (57)	19 (68)	35 (63)		37 (93)	31 (94)	68 (93)		
**ALS diagnostic criteria, n (%)** ^**c**^				0.4199				0.8812	0.2297
Definite	14 (50)	11 (39)	25 (45)		14 (35)	11 (33)	25 (35)		
Probable	14 (50)	17 (61)	31 (55)		26 (65)	22 (67)	48 (66)		
**ALS severity, n (%)** ^**d**^				0.7366				0.3891	0.0468
Grade 1	6 (21)	5 (18)	11 (20)		16 (40)	10 (30)	26 (36)		
Grade 2	22 (79)	23 (82)	45 (80)		24 (60)	23 (70)	47 (64)		
**Mean ALSFRS-R score (SD)**									
Before observation period	43.0 (2.4)	43.4 (2.0)	43.2 (2.2)	0.5085	44.0 (2.4)	43.6 (2.4)	43.8 (2.2)	0.4503	0.0781
Baseline	41.0 (2.5)	41.6 (1.9)	41.3 (2.4)	0.3094	41.9 (2.4)	41.9 (2.4)	42.2 (2.4)	0.3392	0.0247
**Concomitant riluzole, n (%)**	25 (89)	27 (96)	52 (93)	0.2993	37 (93)	29 (88)	66 (90)	0.5045	0.6220

^a^Comparison between FVC_24_ subgroups.

^b^Comparison between treatment arms within each FVC_24_ subgroup.

^c^According to revised El Escorial criteria.

^d^According to Japan ALS severity classification (grade 1–5, with grade 5 being most severe).

ALS, amyotrophic lateral sclerosis; ALSFRS-R, Amyotrophic Lateral Sclerosis Functional Rating Scale-Revised; FVC_24_, forced vital capacity at 24 weeks; SD, standard deviation.

### FVC values in Study 19 and in the FVC_24_ subgroups

In Study 19 overall, the mean (± standard deviation) FVC at baseline was 100.5%p ± 14.97p% in the edaravone group and 97.3%p ± 13.59p% in the placebo group (**Table 3**). At week 24 (end of cycle 6), the mean FVC (FVC_24_) was 87.6%p ± 23.94p% in the edaravone group overall and 80.5%p ± 23.95p% in the placebo group overall (**Table 3**). As expected, the mean FVC_24_ values were lower in the FVC_24_ <80%p subgroups than in the FVC_24_ ≥80%p subgroups (**Table 3**). In particular, the mean FVC_24_ was 60.3%p ± 12.89%p in the placebo FVC_24_ <80%p subgroup. By week 24, 61.5% (40/65) of edaravone subjects and 55.2% (32/58) of placebo subjects maintained FVC ≥80%p (**Table 3**). As expected, by week 48, the mean FVC had decreased in both the FVC_24_ <80%p and the FVC_24_ ≥80%p subgroups (**Table 3**).

**Table 3:** FVC values in the analysis subgroups.

Group	Edaravone	Placebo
**Baseline**		
FAS		
n	69	68
FVC_0_, mean (SD)	100.5%p (14.97%)	97.3%p (13.59%)
**Week 24 (end of cycle 6)**		
FAS^a^		
n	68	66
FVC_24_, mean (SD)	87.6%p (23.94%)	80.5%p (23.95%)
FVC_24_ ≥80%p^b^		
n	40	32
FVC_24_, mean (SD)	103.7%p (16.30%)	97.4%p (12.53%)
FVC_24_ <80%p^c^		
n	25	26
FVC_24_, mean (SD)	66.1%p (8.38%)	60.3%p (12.89%)
**Week 48 (end of cycle 12)**		
FAS^a^		
n	51	36
FVC_48_, mean (SD)	83.9%p (25.00%)	71.8%p (24.17%)
FVC_24_ ≥80%p^b^		
n	37	26
FVC_48_, mean (SD)	93.7%p (20.11%)	80.2%p (19.05%)
FVC_24_ <80%p^c^		
n	14	10
FVC_48_, mean (SD)	58.2%p (17.40%)	50.1%p (23.24%)

^a^LOCF used for subjects who completed cycle 3 (subjects who reached 81 days after the start of treatment).

^b^Data on observed cases in the subgroup with FVC ≥80%p at week 24 (end of cycle 6) (ie, FVC_24_ ≥80%p) in subjects who entered the open-label, active-treatment period.

^c^Data on observed cases in the subgroup with FVC <80%p at week 24 (end of cycle 6) (ie, FVC_24_ <80%p) in subjects who entered the open-label, active-treatment period.

%p, percent of predicted; FAS, full analysis set; FVC_0_, forced vital capacity at baseline; FVC_24_, forced vital capacity at 24 weeks; FVC_48_, forced vital capacity at 48 weeks; LOCF, last observation carried forward; SD, standard deviation.

### Change in ALSFRS-R score in the FVC subgroups

**Fig 3:**
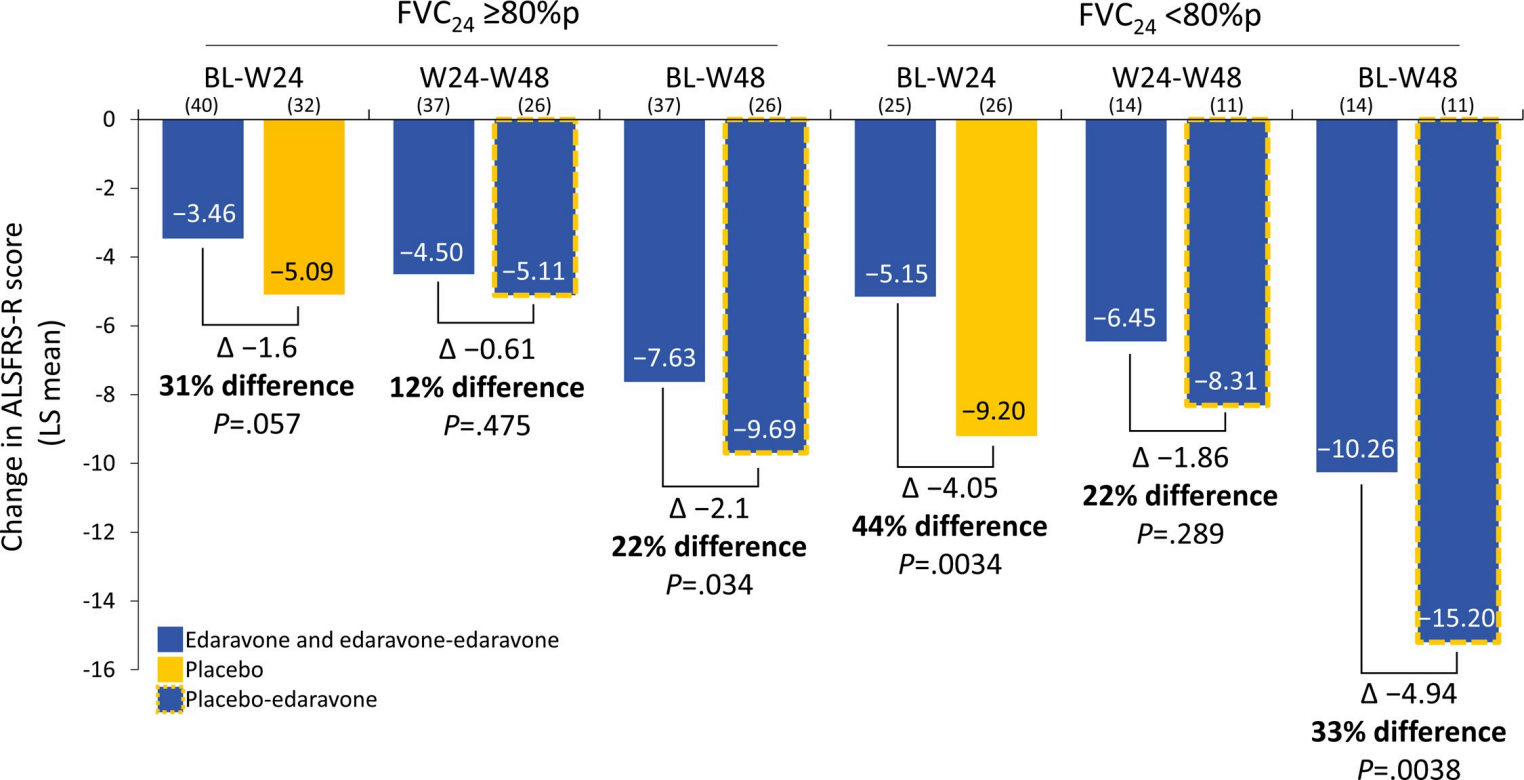
Change in ALSFRS-R scores in FVC_24_ subgroups. Change in ALSFRS-R scores (LS means) in the FVC_24_ ≥80%p and FVC_24_ <80%p subgroups for baseline to week 24 (BL-W24), week 24 to week 48 (W24-W48), or baseline to week 48 (BL-W48) (edaravone, blue columns; placebo, yellow columns; placebo followed by edaravone, blue columns with yellow dashed outlines). A mixed-effects model for repeated measures (MMRM) analysis was conducted on observed cases. LS mean differences and percentage differences between treatment arms are shown. The subject number included in each analysis is shown in parentheses just above each data column. Note that 5 subjects did not have cycle 12 ALSFRS-R data and were not included in the analysis. ALSFRS-R, Amyotrophic Lateral Sclerosis Functional Rating Scale-Revised; BL, baseline; FVC_24_, forced vital capacity in 24 weeks; LS, least squares; W, week.

Significant differences were found among treatment arms through 48 weeks of treatment in change in ALSFRS-R scores in each FVC_24_ subgroup by MMRM analysis (**Fig 3**). For the FVC_24_ ≥80%p subgroup, the changes in ALSFRS-R scores from baseline to week 24 were −3.46 ± 0.55 for edaravone vs −5.08 ± 0.62 for placebo, a difference of 1.61 ± 0.83 (*P* = .057; 95% CI: −0.05, 3.27); from week 24 to week 48, they were −4.50 ± 0.56 for edaravone-edaravone vs −5.11±0.64 for placebo-edaravone, a difference of 0.61±0.85 (*P* = .475; 95% CI: −1.08, 2.3); and from baseline to week 48, they were −7.63±0.62 for edaravone-edaravone vs −9.69 ± 0.72 for placebo-edaravone, a difference of 2.05 ± 0.96 (*P* = .034; 95% CI: 0.16, 3.94). Even more pronounced effects were seen in the FVC_24_ <80%p subgroup. For this subgroup, the changes in ALSFRS-R scores from baseline to week 24 were −5.15 ± 0.94 for edaravone vs −9.20 ± 0.92 for placebo, a difference of 4.05 ± 1.31 (*P* = .0034; 95% CI: 1.41, 6.69); from week 24 to week 48, they were −6.45 ± 1.19 for edaravone-edaravone vs −8.31 ± 1.24 for placebo-edaravone, a difference of 1.86 ± 1.72 (*P* = .289; 95% CI: −1.66, 5.38); and from baseline to week 48, they were −10.26 ± 1.15 for edaravone-edaravone vs −15.20 ± 1.20 for placebo-edaravone, a difference of 4.94 ± 1.67 (*P* = .0038; 95% CI: 1.64, 8.25) (**Fig 3**).

**Fig 4:**
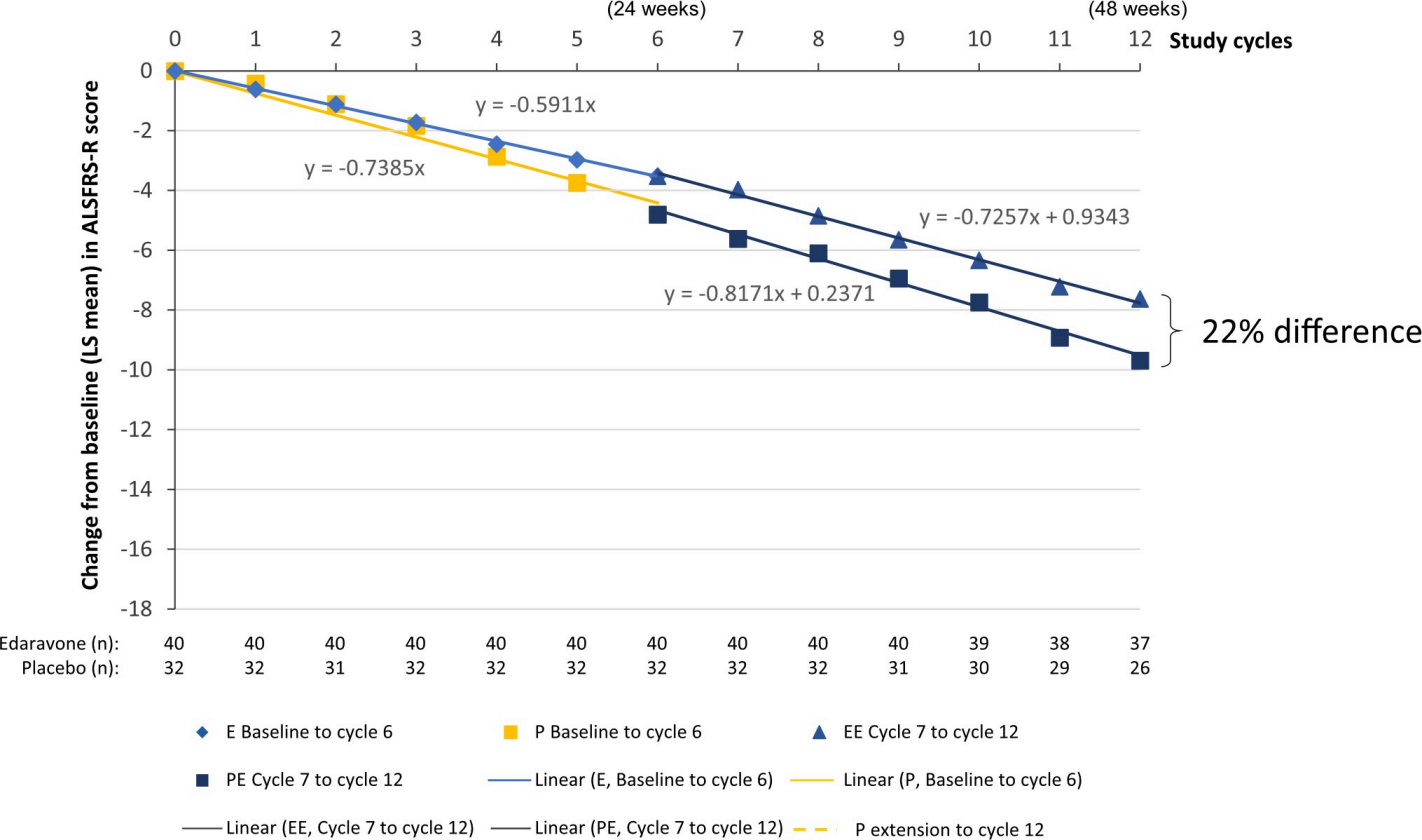
Regression analysis of change from baseline ALSFRS-R scores for the FVC_24_ ≥80%p subgroup. Symbols indicate treatment with edaravone (E, blue diamonds), placebo (P, yellow squares), edaravone-edaravone (EE, blue triangles), or placebo-edaravone (PE, blue squares). Data reflect LS mean change from baseline values. Linear regression line equations are shown on the graph. %p, percent of predicted; ALSFRS-R, Amyotrophic Lateral Sclerosis Functional Rating Scale-Revised; E, edaravone; FVC_24_, forced vital capacity in 24 weeks; LS, least squares; P, placebo.

**Fig 5:**
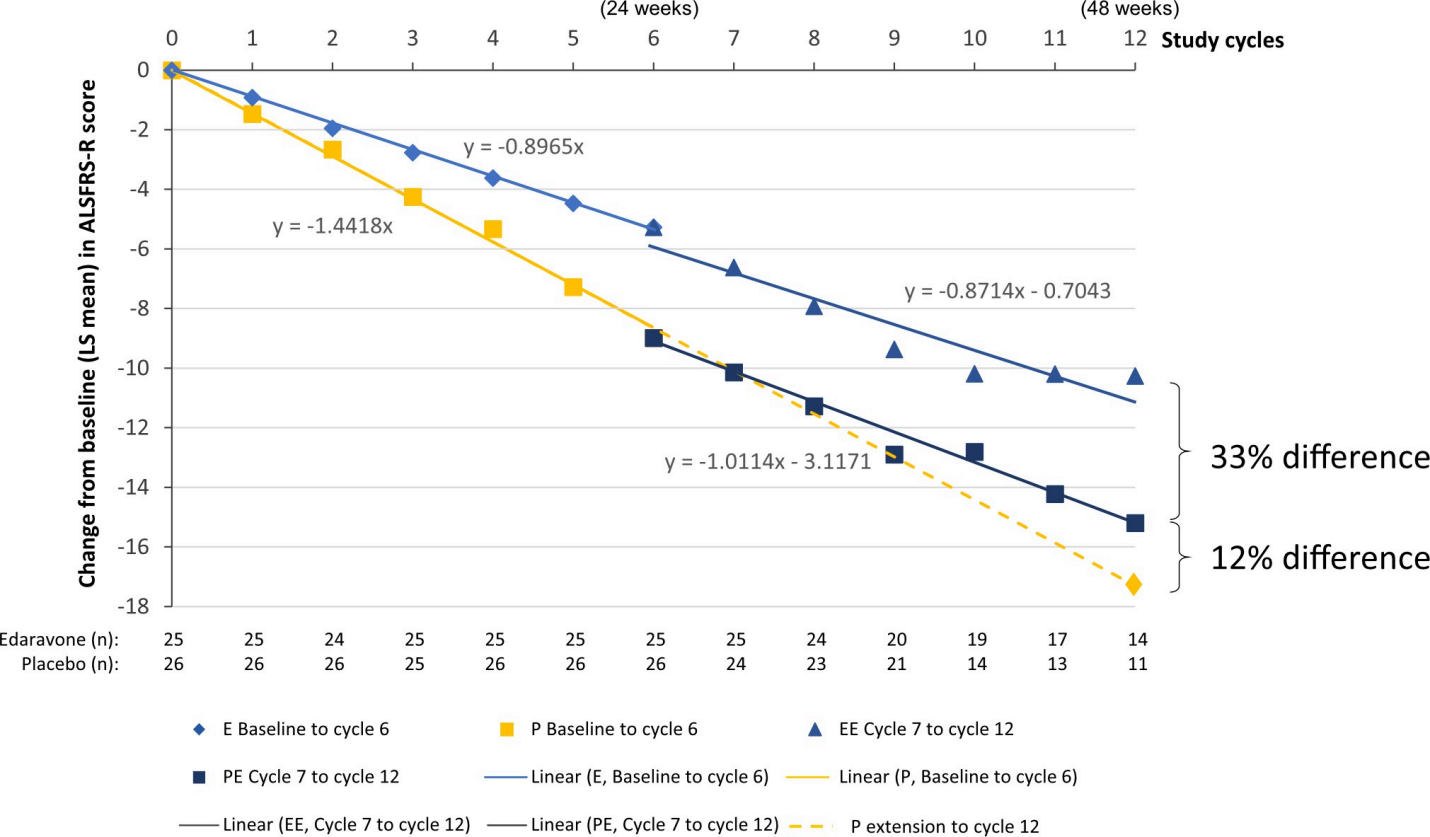
Regression analysis of change from baseline ALSFRS-R scores for the FVC_24_ <80%p subgroup. Symbols indicate treatment with edaravone (E, blue diamonds), placebo (P, yellow squares), edaravone-edaravone (EE, blue triangles), or placebo-edaravone (PE, blue squares). Data reflect LS mean change from baseline values. Linear regression line equations are shown on the graph. ALSFRS-R, Amyotrophic Lateral Sclerosis Functional Rating Scale-Revised; E, edaravone; FVC_24_, forced vital capacity at 24 weeks; LS, least squares; P, placebo.

Linear regression analyses were performed with the subject data from each FVC_24_ subgroup in each phase of the study (**Figs 4 and 5**). As expected, the slopes of the edaravone and edaravone-edaravone linear regression lines were similar to one another in each subgroup, indicating that edaravone had a similar effect on ALSFRS-R score during the first 24 weeks of the study as well as from week 24 through 48 (**Figs 4 and 5**). The placebo subjects from the FVC_24_ <80%p subgroup demonstrated a statistically significant change in slope in ALSFRS-R after starting edaravone therapy at week 24 (*P* = .006, linear mixed-effect model) (**Fig 5**). From the linear regression analysis, the change from baseline in ALSFRS-R score at week 48 for the placebo-edaravone subjects was –15.20, as compared with a projected value of –17.3 if the subjects had remained on placebo, based on linear regression of the placebo arm, a difference of 12%.

**Fig 6:**
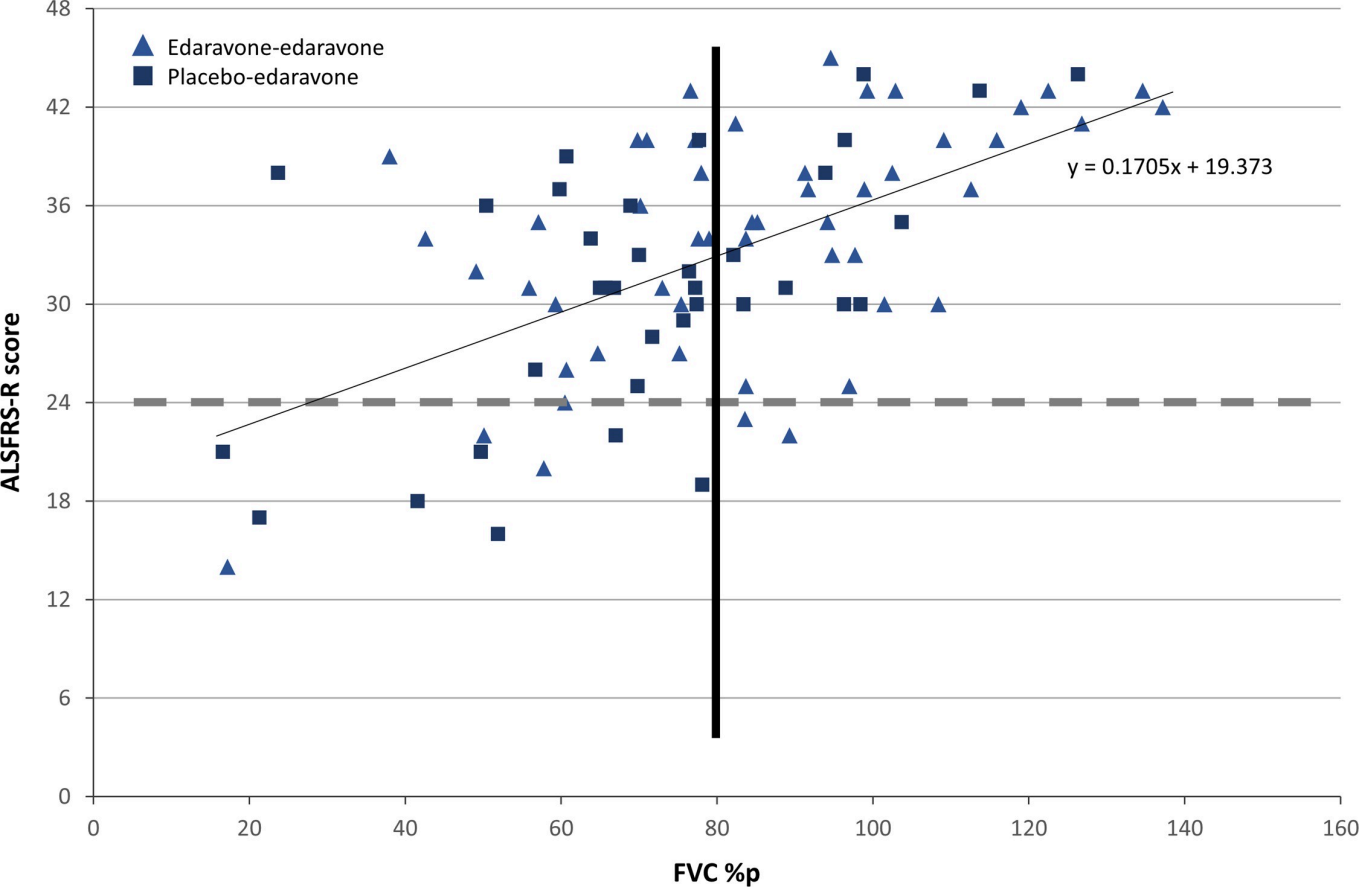
ALSFRS-R score vs FVC at week 48. Graph of ALSFRS-R vs FVC values at week 48. The dashed gray horizontal line delineates an ALSFRS-R score of 24; the black vertical line delineates an FVC value of 80%p. Each triangle represents 1 subject in the edaravone-edaravone group and each square represents one subject in the placebo-edaravone group at week 48. Over a wide range of FVC_48_%p, a large number of ALS subjects participating in the clinical trial maintained an ALSFRS-R total score at or above 24 throughout the clinical trial period. Linear regression line equation is shown on the graph. %p, percent of predicted; ALS, amyotrophic lateral sclerosis; ALSFRS-R, Amyotrophic Lateral Sclerosis Functional Rating Scale-Revised; FVC, forced vital capacity.

The analysis of the ALSFRS-R score vs FVC at week 48 indicated that, at this time point, most subjects in the study had ALSFRS-R scores >24, including those with FVC at 48 weeks <80%p (**Fig 6**), indicating that, despite a decline in respiratory function as measured by FVC, these subjects appeared to have functionality in other domains of the ALSFRS-R (eg, gross motor, fine motor, and bulbar domains). These observations confirm that ALS subjects having decreased vital capacity on placebo, receiving delayed edaravone, may still benefit from a treatment that slows the loss of physical function.

## Discussion

Edaravone treatment was associated with significantly less functional decline, as measured by ALSFRS-R score, in subjects in both the FVC_24_ ≥80%p subgroup and the FVC_24_ <80%p subgroup. Several lines of evidence indicate that edaravone was effective in study subjects with FVC <80%p. For example, in the FVC_24_ <80%p subgroup, during the double-blind treatment period, edaravone was associated with a 44% reduction in ALSFRS-R score loss compared with placebo (a change in ALSFRS-R score of −5.15 for edaravone vs −9.20 for placebo; *P* = .0034). Moreover, from baseline to week 48, edaravone was associated with a 33% reduction in ALSFRS-R score loss compared with placebo (a change in ALSFRS-R score of −10.26 for edaravone-edaravone vs −15.20 for placebo-edaravone; *P* = .0038). In addition, the placebo subjects from the FVC_24_ <80%p subgroup demonstrated a notable change in slope in ALSFRS-R after starting edaravone therapy at week 24, indicating that edaravone slowed the rate of disease progression, as measured by ALSFRS-R, in subjects with a mean FVC well below 80%p (mean FVC_24_ = 60.3%p ± 12.89%). Based on this post-hoc analysis, we conclude that edaravone shows a statistically significant reduction in the rate of disease progression in subjects with FVC <80%p, providing benefit for ALS subjects in both the FVC_24_ ≥80%p subgroup and the FVC_24_ <80%p subgroup.

While the inclusion criteria for Study 19 were based on a post-hoc analysis of the first phase 3 study, Study 16 (MCI186-16), where investigators reasoned that subjects with respiratory dysfunction might show rapid progression masking an effect of an active treatment, such as edaravone, resulting in the criterion in Study 19 requiring FVC ≥80%p with a baseline score of 4 on all ALSFRS-R respiratory items [11], our data suggest that edaravone was effective in study subjects with both FVC ≥80%p and FVC <80%p.

The FDA-approved indication for Radicava^®^ (edaravone) is “for the treatment of ALS” [20]. The findings of this current post-hoc analysis support the FDA-approved labeling that edaravone may be of benefit to the general ALS patient population, in terms of slowing the rate of loss of function, as measured by ALSFRS-R, independent of the respiratory status, as measured by FVC%p at the start of edaravone therapy.

Study 16 and Study 19 have been the only randomized, controlled, phase 3 studies of edaravone in patients with ALS. Because of the interest in the use of this drug in ALS, retrospective analyses and literature reviews have recently been published. One retrospective analysis conducted with patients from the Veterans Health Administration system in the United States indicated that patients receiving edaravone treatment may have had increased hospitalization events [21]. However, in that study, patients receiving edaravone were older and had a longer disease duration than the comparator group of patients. In that study, edaravone may have been associated with a lower death rate, although the difference was not statistically significant with the numbers of patients included in the study. A recent literature review of additional retrospective or observational studies with edaravone found that some of the studies supported the benefit of edaravone in slowing of disease progression (eg, studies in Asian countries), while other studies were not conclusive (including studies in Europe and Israel) [22]. This literature review indicated that edaravone was well tolerated in all studies reviewed.

As a post-hoc, subgroup analysis of Study 19, this study is subject to the limitations inherent in post-hoc analyses. For example, these post-hoc analyses were not prespecified in Study 19. In addition, there were smaller sample sizes in each subgroup as the trial progressed and there was a lack of control for type 1 error and no adjustments for multiplicity in these post-hoc analyses. An MMRM analysis and multiple regression analyses were selected to investigate the efficacy of edaravone in the 2 FVC_24_ subgroups. This approach showed consistent results between the methods employed and between actual treatment vs projected treatment; however, careful consideration with regard to study limitations is needed when interpreting the results. Heterogeneity in the disease phenotype and nonlinearity of disease progression at different disease epochs needs to be considered [23]. Nevertheless, the tendency for ALS subjects to remain on edaravone treatment (Fig 2) provides additional support to the conclusions presented.

One of the proposed mechanisms of action of edaravone is the reduction of oxidative stress [24]. Patients with ALS are known to have elevated levels of oxidative stress biomarkers and reductions in antioxidant enzymes [25, 26]. In addition, it has been postulated that respiratory failure in patients with ALS may increase oxidative stress [27]. Thus, one of the potential mechanisms of action of edaravone in slowing the loss of physical function in patients with FVC <80%p may be due to the hypothesized antioxidant effects of this therapy.

Modifying the types of statistical modeling applied to ALS clinical trials has provided insights to the translation of the treatment effects to different patient populations. For riluzole, that is the first treatment identified to improve survival in ALS patients, differential beneficial treatment effects have been reported in traditional subgroup analyses [28], statistical learning approaches for “automated” subgroup analysis [29], and estimation of individualized treatment effects [30]. A recent review of real-world evidence regarding riluzole treatment earlier in the course of ALS than originally studied in the pivotal clinical trials identified a significantly larger treatment effect than that reported in the original clinical trials [31]. Furthermore, the survival benefit of riluzole in observational studies is statistically significant in those patients with FVC ≥80%p and FVC <80%p [32]. Edaravone now is shown to have an effect on function, measured with the ALSFRS-R, that is present in riluzole-treated ALS patients who have FVC ≥80%p and FVC <80%p.

## Conclusions

Post-hoc analysis of edaravone Study 19 data through week 48 (ie, 24-week double-blind followed by 24-week open-label edaravone treatment) indicated that for ALS subjects in both the FVC_24_ ≥80%p and FVC_24_ <80%p subgroups edaravone provided a reduction in ALSFRS-R score loss compared with placebo. Subjects in the FVC_24_ <80%p placebo subgroup responded to edaravone treatment as demonstrated by a change in slope of the ALSFRS-R score-vs-time graph after starting edaravone treatment (placebo-edaravone arm). This analysis provides evidence that edaravone may have benefit in ALS patients, irrespective of whether they start treatment when their FVC is ≥80%p or <80%p.
